# Gastro-intestinal lesions are not relatable to diarrhoea or specific pathogens in post-weaning diarrhoea (PWD) in pigs

**DOI:** 10.1186/s13028-023-00693-y

**Published:** 2023-07-03

**Authors:** Sophie Amalie Blirup-Plum, Henrik Elvang Jensen, Søren Saxmose Nielsen, Karen Pankoke, Mette Sif Hansen, Ken Steen Pedersen, Esben Østergaard Eriksen, Jens Peter Nielsen, John Elmerdahl Olsen, Egle Kudirkiene, Lars Erik Larsen, Nicole Bakkegård Goecke, Kristiane Barington

**Affiliations:** 1 Department of Veterinary and Animal Sciences, University of Copenhagen, Grønnegårdsvej 7, 1870 Frederiksberg C, Denmark; 2 Ø-Vet A/S, 4700 Næstved, Denmark

**Keywords:** Histopathology, Microbiology, Pigs, Post-weaning diarrhoea

## Abstract

**Background:**

Post-weaning diarrhoea (PWD) is a multifactorial condition and the most well documented infectious cause is enterotoxigenic *Escherichia coli*. The objective of the study was to investigate possible associations between pathological manifestations and pathogens in pigs with and without PWD. The study was conducted as a case–control study and included a total of 173 pigs from 9 different commercial intensive indoor production herds in eastern Denmark.

**Results:**

Based on clinical examination, a total of 89 piglets with PWD (cases) and 84 piglets without PWD (controls) were included. Most of the pigs (n = 105/173) presented gastric lesions, which were more frequently observed in the control group. The odds of gastric ulcers were lower among pigs with PWD compared to pigs without PWD with an odds ratio (OR) of 0.2 (0.0; 0.7). Abnormal content in the colon was associated with PWD, with an OR of 6.5 (3.2; 14.3). No apparent association was found between lesions and the various pathogens or a combination of these. The odds of neutrophilic granulocyte infiltration were lower in the jejunum among pigs with PWD (OR 0.3 [0.1; 0.6]) compared to pigs without PWD. The association between neutrophilic granulocyte infiltration in jejunum and PWD differed between the herds (P = 0.03). Furthermore, the associations between PWD and hyperleukocytosis (P = 0.04) or infiltration of eosinophilic granulocytes (P = 0.04) in ileum were also herd dependent. Histopathology revealed several lesions not relatable to PWD.

**Conclusion:**

The association between lesions and specific pathogens or PWD is more complex than anticipated.

**Supplementary Information:**

The online version contains supplementary material available at 10.1186/s13028-023-00693-y.

## Background

Post-weaning diarrhoea (PWD) is a multifactorial condition occurring within the first 14 days after weaning [1–3]. PWD has mainly been associated with the presence and proliferation of enterotoxigenic *Escherichia coli* (ETEC) [1, 4, 5], however, ETEC may not be demonstrated in pigs with clinical signs [1, 5]. Therefore, it has been suggested that other causative factors, such as gut microbiota dysbiosis [6] and protein fermentation [3, 7] may cause inflammation of the intestines and diarrhoea in newly weaned pigs.

PWD compromises animal welfare and is a major cause of death [5, 8]. High pig mortality reduces productivity and causes substantial economic losses [8, 9]. Endemic disease further leads to increased use of antimicrobials, which adds to the concern of antimicrobial resistance [8, 9]. In Denmark, 76% of the veterinary-prescribed antimicrobials is used in the pig sector [10]. The antimicrobial strategy is considered to be metaphylactic and is dominated by batch medication [11, 12]. In the years 2002–2012, approximately 75% of the antimicrobial use in Danish nursery pigs was prescribed due to gastro-intestinal diseases [13]. Therefore, it is important to clarify the causes of disease to avoid unnecessary treatment with antimicrobials as some conditions will not benefit from antimicrobial treatment. Moreover, multiple enteric infections may occur consecutively, resulting in more complex clinical patterns [8, 12, 14]. Another concern is subclinical infection caused by, e.g., *Lawsonia intracellularis* [15, 16], which however is more frequently seen in growing to finishing pigs [17]. The features of sub-clinical infection, e.g., declined growth rate, affects production economics and the affected pigs may further spread potential pathogens to other pigs [16]. Therefore, an accurate diagnostic approach for PWD, caused by e.g., *E. coli,* would be to combine clinical signs, necropsies and laboratory procedures such as histopathology and quantitative polymerase chain reaction (qPCR) [5]. Histopathology is considered critical, not only to investigate lesions and dysfunctions, but also to confirm the impact of the various pathogens in combined infections as well as infections with a single causative agent [8].

The objectives of the present study were to investigate possible associations between gastro-intestinal pathological manifestations and PWD and the presence of known pathogens of enterocolitis, respectively. In addition, it was investigated if intestinal lesions might be caused by an interaction between viral and bacterial pathogens. The study was designed in order to mimic the practical conditions of a field survey and was kept as a primarily descriptive one with the limitations incurred by this.

## Methods

### Study design

The study was conducted in May–October 2019 as a case–control study. Through public register data, a total number of 9 commercial intensive indoor production herds that weaned pigs at approximately three to four weeks of age without adding medicinal zinc to the food were identified. All herds were located in eastern Denmark (i.e. the Islands Amager, Falster, Funen, Lolland and Zealand, and the Triangle region in Jutland) within 2.5 h driving range from the necropsy facility. The herds were included as they suffered from recurrent outbreaks of PWD, and subsequently initiated antimicrobial batch medication. The herds were visited on the day, where the herd personnel wanted to initiate antimicrobial batch medication. At each outbreak, faecal samples were collected (by EØE and technicians) from a systematic random sample of approximately 100 pigs. The faecal samples were assessed at gross inspection and scored on the four-point scale described by Pedersen and Toft [18]. In brief, firm faeces (score 1) and soft but shaped faeces (score 2) were considered non-diarrhoeic and therefore eligible as controls; loose faeces (score 3), and watery faeces (score 4) were considered diarrhoea and therefore eligible as cases (PWD) [18]. Up to 10 cases of PWD was randomly selected among all pigs suffering from diarrhoea in each herd. Likewise, up to 10 controls were randomly selected among all the pigs without apparent PWD. In two herds, less than 10 apparent cases (n = 4 in herd C) or controls (n = 9 in herd G) were available. Here all pigs matching the cases/control definition were included, but it caused an unequal number of cases (n = 84) and controls (n = 89) in the final dataset. On the day of the clinical examination and sampling for microbiology, all included control and case pigs larger than 5 kg body weight were stunned using a captive bolt. As it was dangerous (for both pigs and technicians) to use the captive bolt for small pigs less than 5 kg of body weight (n = 22), pigs were stunned by performing a forceful blunt trauma to their forehead against the concrete floor. Both stunning methods were followed by exsanguination by incision of *vena jugularis* and *arteria carotis*. The euthanized pigs were transported to the necropsy facilities by car, stored over night at 5 °C, and necropsied the next morning.

### Microbiology

The clinical recordings of the pigs and grouping (case versus control) were blinded to the persons conducting the laboratory diagnostics. A rectal sterile cotton swab sample was collected from all pigs and immediately placed in a plastic tube with 5 mL sterile phosphate buffered saline (PBS) solution. The tubes were placed in ice water during the rest of the herd visit and in a cooling box with ice cubes during the transport to the university. At arrival to the university (approx. 1–8 h after sampling), the samples were stored at 4 °C until processing the next day.

#### Culture-based methods

Culture based methods aimed to detect ETEC, *Clostridium perfringens*, and *Salmonella enterica*.

ETEC diagnostic was performed essentially as previously described by Eriksen et al. [1] using culturing on blood agar (Oxoid, CM0055, Thermofisher Scientific, Denmark). For *E. coli,* a semi-quantitative approach was used to score haemolytic colonies as absence (no haemolytic *E. coli*), low (haemolytic *E. coli* making up < 50% of colonies on the plate), or dominating (haemolytic *E. coli* making up > 50% of colonies on the plate)*. E. coli* classification was confirmed by matrix-assisted laser desorption ionisation time-of-flight (MALDI-TOF) mass spectrometry identification with VITEK® MS RUO instrument (bioMérieux, Marcy l’Etoile, France) and CHCA matrix solution (VITEK® MS-CHCA, bioMérieux SA), and polymerase chain reaction (PCR) genotyping for virulence factors. PCR methods were directed against F4, F5, F6, F41 fimbriae, LT, STa, STb enterotoxins, and verotoxins 1, 2 and 2e, using published primer sequences [19–21]. ETEC in the current study was defined as *E. coli* expressing at least one of the fimbriae and one enterotoxin. Results from semi-quantitative determination of ETEC were missing for 20 pigs (two herds), and in these instances we made imputations based on the cycle threshold (Ct) values for *E. coli* fimbria antigens generated by qPCR (described below).

For detection of *C. perfringens*, a loop full (1 µL) of the rectal swab PBS solution was inoculated on blood agar (Oxoid, CM0055, Thermofisher Scientific, Denmark) with 5% calf blood and incubated at 37 °C for 48 h at anaerobic conditions. Double-haemolytic colonies were selected, and a multiplex PCR identified the toxin-type of isolates, as described elsewhere [22]. This multiplex PCR method allows identification of the four major toxin types of *C. perfringens* in addition to those encoding the enterotoxin. No enrichment for spores was conducted.

For isolation of *S. enterica*, 100 μL of the faecal PBS solution was enriched in 9 mL of BPW overnight at 37 °C, and 100 μL was then spotted onto Modified Semisolid Rappaport Vassiliadis agar (Oxoid CM0910) and incubated aerobically at 37 °C for 24 h. Typical *Salmonella enterica* colonies, i.e., those forming a grey-white, turbid zone extending out from the inoculated drop, were purified on blood agar, and the species identity was confirmed by MALDI-TOF. Serotype was determined by whole genome sequencing as previously described [23].

#### High-throughput real-time PCR for identification of E. coli *F4,* E. coli *F18,* L. intracellularis, Brachyspira pilosicoli, *Porcine circovirus type 2 (PCV2), and rotavirus A.*

Prior to high-throughput real-time PCR analysis, nucleic acids were extracted from the rectal swab samples using the extraction robot QIAcube HT (QIAGEN, Hilden, Germany) and the Cador Pathogen 96 QIAcube HT kit (Indical Bioscience, Leipzig, Germany) using the manufacturer’s instructions. Before nucleic acid extraction, rectal swab samples were prepared by vortexing and centrifuging 400 μL of each individual sample, and 200 μL of the supernatant was subsequently used for extraction. Positive and negative (Invitrogen™ UltraPure™ DNase/RNase-Free Distilled Water, Thermofisher, Denmark) controls were included in each extraction. The nucleic acids were stored at − 80 °C until further analysis. Extracted rectal swab samples were reverse transcribed/pre-amplified (RNA targets) and pre-amplified (DNA targets) as follows: For RNA targets, reverse-transcription and pre-amplification were performed in a final volume of 15 μL (AgPath-ID one-step reverse transcriptase (RT)-PCR reagents kit; Applied Biosystems), in which 7.5 μL of 2 × RT-PCR buffer was mixed with 1.00 μL of primer mix (200 nM, containing all primers listed in Additional file 1: Table S1), 0.6 μL of 25 × RT-PCR enzyme mix, 2.90 μL of nuclease-free water, and 3 μL of RNA. One-tube combined reverse-transcription and pre-amplification was performed (ProFlex PCR System; Applied Biosystems) with the following thermal cycling conditions: 20 min at 45 °C, 10 min at 95 °C, followed by 24 cycles at 94 °C for 15 s, and 60 °C for 45 s. The pre-amplified complementary DNA (cDNA) was stored at − 20 °C. For pre-amplification of DNA targets, master mix (TaqMan PreAmp; Applied Biosystems) was used. The reaction was performed in a final volume of 10 μL containing 5 μL of master mix, 2.5 μL of primer mix (200 nM, containing all primers), and 2.5 μL of DNA. Pre-amplification was performed (ProFlex PCR System; Applied Biosystems) with the following thermal cycling conditions: 95 °C for 10 min, followed by 14 cycles at 95 °C for 15 s, and 60 °C for 4 min. The pre-amplified DNA was stored at − 20 °C. The rectal swabs were tested by high-throughput real-time PCR amplification, using the BioMark HD (Fluidigm, South San Franscisco, CA, USA) and 192.24 dynamic array integrated fluidic circuit system (Fluidigm) as previously described [24, 25]. Simultaneous real-time PCR reactions were applied for identification of the following enteral pathogens; *E. coli* F4, *E. coli* F18, *L. intracellularis*, *Brachyspira pilosicoli*, Porcine circovirus genotype 2 (PCV2), and rotavirus A as previously described [24].

### Necropsy

The clinical recordings and grouping (case versus control) were blinded to the pathologists. The pigs were placed on their backs and the nutritional status was recorded. Afterwards, the abdominal cavity was opened with an incision through the skin, muscles and fascias from the *processus xiphoideus* caudally to the cranial margin of the pelvis. The intestines and stomach were eviscerated and examined. The stomach was separated from the intestines and cut open along the greater curvature. The content was removed and inspected, and the mucosa examined for e.g., ulcers and hyperkeratosis. Segments from the middle of jejunum, ileum, and colon were cut open along the attachment of the mesentery, and the intestinal content, intestinal wall, and the mucosa were examined. The content was recorded as normal or abnormal (watery, slimy, firm/compact, and/or haemorrhagic). The intestinal wall was recorded as normal or thickened. The intestinal mucosa was recorded as normal or abnormal (ulcers, haemorrhage, hyperaemia, and dry).

### Histopathology

Samples from midst sections of the jejunum, ileum and colon were pinned on styrofoam and immersion-fixed in 10% neutral buffered formalin for approximately three days. Following trimming, the tissues were processed through graded concentrations of ethanol (75%, 80%, 96% and 99% ethanol) and xylene (pure), and embedded in paraffin. Afterwards, tissue sections of 4–5 µm were stained with haematoxylin and eosin (HE).

To increase validation, all histological evaluations were carried out blinded using a Leica DMLB microscope. The histological sections from the jejunum, ileum and colon were examined for lesions. Lesions were registered as present or absent except for crypt abscesses, which were scored on a semi-quantitative scale: (0) absence of crypt abscesses, (1/few) 1–4 crypt abscesses, (2/abundant) 5 + crypt abscesses. The scoring was carried out in the area with the highest number of crypt abscesses using a 10 × objective and all crypts were counted regardless of their orientation. The infiltration of eosinophilic granulocytes was defined by a cut-off of ≥ 30 eosinophilic granulocytes per high power field (40 × objective). Neutrophilic granulocyte infiltration was defined by a cut-off of ≥ 5 neutrophilic granulocytes per high power field. The scoring was carried out in the areas with highest density of eosinophilic granulocyte and neutrophilic granulocytes, respectively. Mononuclear cell infiltration of 15 or more mononuclear cells, i.e., infiltration of macrophages, monocytes, lymphocytes and plasma cells, was defined as an accumulation. Finally, the sections were examined for the presence of protozoa.

### Immunohistochemistry (IHC)

For immunostaining, the tissue sections were mounted on adhesive glass slides (Epredia, Portsmouth, NH, USA).

#### Crypt abscesses

IHC based on MAC387 (Serotec MCA874G, diluted 1:1000), staining of macrophages and NGs, and cytokeratin staining (Mouse anti-cytokeratin clone AE1/AE3, DAKO M3515, diluted 1:1200) were used as previously described [26–28] on various intestinal sections from selected pigs with crypt abscesses in order to confirm the composition of the debris.

#### PCV2

All intestinal sections from all animals positive for PCV2 by high-throughput real-time PCR were IHC stained for PCV2. A mouse monoclonal anti-PCV2 capsid was used (GT863, Nordic biosite/Genetex GTX634210, 1 mg/mL). The immunostaining technique was performed by application of the Ultravision ONE Detection system horseradish peroxidase (HRP) (Epredia, TL-125-HLJ, Breda, Netherlands). First, the sections were dewaxed. This was followed by blocking of endogenous peroxidase activity by 3% H_2_O_2_ (50 mL methanol added to 50 mL 6% H_2_O_2_) for 15 min, heat-induced epitope retrieval by citrate buffer (pH 6.0) for 2 × 5 min (100 °C), and blocking of unspecific binding by Ultra Protein Block for 5 min (Epredia, TA-125-PBQ, Breda, Netherlands). The tissue sections were then incubated with the primary antibody GT863 (diluted 1:300 in tris-buffered saline (TBS)) for approximately 20 h at 4 °C. Ultravision HRP polymer was added for 30 min and AEC vector (AEC substrate kit SK-4200, Vector Laboratories, Inc, Newark, CA, USA) for 10 min. Throughout the protocol, with the exception of the step between Ultra Protein blocking of unspecific binding and the application of the primary antibody, slides were washed in TBS, pH 7.6. Counterstaining was done in Mayer’s haematoxylin (AMPQ002454.5000, VWR international) and the sections were rinsed in distilled water. Coverslips were mounted with glycerol-gelatine. Positive and negative controls were run on a PCV2 positive lymph node. Negative controls included substitution of the primary antibody with a nonsense antibody (IgG1, XO931, diluted 1:66 in TBS).

#### Lawsonia intracellularis

The intestinal sections from the jejunum, ileum and colon from two pigs in each herd were chosen for IHC staining towards *L. intracellularis.* The selected animals presented lesions similar to those caused by the bacterium, i.e., hyperplastic crypts and necrosis. A mouse monoclonal antibody (mAb) (Law1-DK / BIO 323, Bio-X Diagnostics) and a method modified from Jensen et al. [17] was used. Modifications included; (1) replacement of EnVision + TM with detection system horseradish peroxidase (HRP) (Epredia, TL-125-HLJ, Breda, Netherlands) prior to mAb, and (2) AEC vector was replaced with DAB substrate kit (Cell Marque 957D-40 500 mL kit). Positive and negative controls were run on a *Lawsonia*-positive ileum from a pig. Negative controls included substitution of the primary antibody with a nonsense antibody (IgG1, X0931) diluted 1:3114 in TBS).

### Statistics

A descriptive analysis using summary statistics was performed to associate pathological findings to clinical and microbiological results, respectively. Odds ratio (OR) and associated 95% confidence intervals (CI) were estimated using median-unbiased estimation (mid-p) for gross pathology and histological findings, respectively, among pigs with diarrhoea compared to pigs without diarrhoea, while using the epitools-package [29] in R version 4.1.2 [30]. Regardless of a small sample size, a logistic regression model was used to investigate possible differences in associations between investigated variables (neutrophilic granulocyte infiltration, hyperleukocytosis, eosinophilic granulocyte infiltration, hyperplastic crypts, and crypt abscesses) and PWD between herds. The objective for these herd-specific analyses were to identify if herd-specific outcomes prevailed and could explain possible differences. Level of significance was set at P ≤ 0.05. All calculations and analyses were executed using R.

## Results

Based on the clinical examinations, the study included 89 cases of PWD and 84 controls without PWD.

### Microbiology

Results from the analyses of the rectal swaps demonstrated *S. enterica* serotypes *Typhimurium (S. Typhimurium*) and *Derby *(*S. Derby*), ETEC*,* haemolytic *E. coli* (non-ETEC)*, C. perfringens,* rotavirus A, and PCV2. *B. pilosicoli* and *L. intracellularis* were not detected in any of the pigs. The total number of pathogen positive pigs and their distribution of lesions within the different intestinal regions are shown in Tables 1 and 2. A pig was noted as positive for a specific pathogen when tested positive by either PCR and/or bacterial culturing. The majority of combinations of pathogens could not be further investigated as there were too few positive pigs in the various groups of combinations. Only pigs positive for haemolytic *E. coli* (non-ETEC) in combination with rotavirus A and pigs positive for ETEC (> 50% haemolytic colonies) in combination with rotavirus A were qualified for further analyses (minimum of 20 cases and/or controls in total).

**Table 1:** Histopathological manifestations in the jejunum, ileum and colon and the various pathogens in pigs with post-weaning diarrhoea (PWD)

Histopathology Pathogen (*n*)	Jejunum (*n*)									Ileum (*n*)				
	Crypt abscesses (abundant/few)	Dilated crypts	Eosinophilic granulocyte infiltration	Haemorrhage	Hyperleukocytosis	Hyperplastic crypts	Hypersecretion	Necrosis	Neutrophilic granulocyte infiltration	Crypt abscesses (abundant/few)	Dilated crypts	Eosinophilic granulocyte infiltration	Haemorrhage	Hyperleukocytosis
*Clostridium perfringens* type C (2)	0/0	0	1	0	0	0	0	0	0	0/0	1	2	0	0
*Escherichia coli (E. coli) –* haemolytic non-enterotoxigenic *E. coli* (24*^a^)	0/2	0	8	0	3	0	0	0	1	0/9	9	19	1	6
Enterotoxigenic *E. coli,* < 50% haemolytic colonies (7*^b^)	0/1	0	2	0	0	1	0	0	1	1/3	4	5	0	2
Enterotoxigenic *E. coli*, > 50% haemolytic colonies (24*^c^)	0/4	0	8	0	4	0	0	0	3	2/7	11	18	0	7
Enterotoxigenic *E. coli, all* (31*^d^)	0/5	0	10	0	4	1	0	0	4	3/10	15	23	0	9
Porcine Circovirus type 2 (1)	0/0	0	0	0	1	0	0	0	0	0/0	0	1	0	0
Rotavirus A (78^e^)	0/8	0	34	0	11	2	0	0	5	5/28	34	62	2	17
*Salmonella* spp (10)	0/2	0	5	0	1	0	0	0	0	2/3	5	8	0	3

Histopathology Pathogen (*n*)	Ileum (*n*)				Colon (*n*)								
	Hyperplastic crypts	Hypersecretion	Necrosis	Neutrophilic granulocyte infiltration	Crypt abscesses (abundant/few)	Dilated crypts	Eosinophilic granulocyte infiltration	Haemorrhage	Hyperleukocytosis	Hyperplastic crypts	Hypersecretion	Necrosis	Neutrophilic granulocyte infiltration
*Clostridium perfringens* type C (2)	1	1	0	0	0/1	1	0	0	0	2	1	0	0
*Escherichia coli (E. coli) –* haemolytic non-enterotoxigenic *E. coli* (24*^a^)	10	9	0	2	2/9	8	0	0	3	20	8	0	3
Enterotoxigenic *E. coli,* < 50% haemolytic colonies (7*^b^)	2	4	0	0	0/4	3	0	0	0	5	3	0	1
Enterotoxigenic *E. coli*, > 50% haemolytic colonies (24*^c^)	15	11	2	4	7/12	9	0	1	2	21	8	0	4
Enterotoxigenic *E. coli, all* (31*^d^)	17	15	2	4	7/16	12	0	1	2	26	11	0	5
Porcine Circovirus type 2 (1)	1	0	0	0	0/0	1	0	0	0	1	1	0	0
Rotavirus A (78^e^)	43	34	2	11	13/30	30	0	1	4	70	29	0	11
*Salmonella* spp (10)	7	5	0	1	4/3	2	0	1	1	8	2	0	2

For each pathogen, the total number of positive pigs is listed in the parenthesis in the first column. Moreover, the numbers of pigs with specific lesions within the different intestinal regions are presented for each of the pathogens

n: Number of positive pigs

*Seven animals were excluded due to unknown ETEC status

^a^One out of the 24 pigs had missing values in several of the variables due to autolysis

^b^One of the seven pigs had missing values in several of the variables due to autolysis

^c^One of the 24 pigs had missing values in several of the variables due to autolysis

^d^Two of the 31 pigs had missing values in one or more of the variables due to autolysis

^e^Six of the 78 pigs had missing values in one or more variables due to autolysis

**Table 2:** Histopathological manifestations in the jejunum, ileum and colon and the various pathogens in pigs without post-weaning diarrhoea (PWD)

Histopathology Pathogen (*n*)	Jejunum (*n*)									Ileum (*n*)				
	Crypt abscesses (abundant/few)	Dilated crypts	Eosinophilic granulocyte infiltration	Haemorrhage	Hyperleukocytosis	Hyperplastic crypts	Hypersecretion	Necrosis	Neutrophilic granulocyte infiltration	Crypt abscesses (abundant/few)	Dilated crypts	Eosinophilic granulocyte infiltration	Haemorrhage	Hyperleukocytosis
*Escherichia coli (E. coli) –* haemolytic non-enterotoxigenic *E. coli* (24*^a^)	0/3	0	9	0	1	2	0	0	4	0/11	8	17	0	2
Enterotoxigenic *E. coli,* < 50% haemolytic colonies (9*^b^)	0/1	0	6	0	0	0	0	0	1	0/4	4	7	0	0
Enterotoxigenic *E. coli,* > 50% haemolytic colonies (11*)	0/1	0	4	0	2	0	0	0	3	0/5	4	8	0	2
Enterotoxigenic *E. coli,* all (20*^c^)	0/2	0	10	0	2	0	0	0	4	0/9	8	15	0	2
Porcine Circovirus type 2 (5)	0/0	0	0	0	0	0	0	0	1	0/2	2	3	0	1
Rotavirus A (69^d^)	0/5	4	35	1	4	3	4	0	14	1/33	27	53	0	12
*Salmonella* spp (6)	0/1	1	5	1	2	1	1	0	0	0/3	3	5	0	0

Histopathology Pathogen (*n*)	Ileum (*n*)				Colon (*n*)								
	Hyperplastic crypts	Hypersecretion	Necrosis	Neutrophilic granulocyte infiltration	Crypt abscesses (abundant/few)	Dilated crypts	Eosinophilic granulocyte infiltration	Haemorrhage	Hyperleukocytosis	Hyperplastic crypts	Hypersecretion	Necrosis	Neutrophilic granulocyte infiltration
*Escherichia coli (E. coli) –* haemolytic non-enterotoxigenic *E. coli* (24*^a^)	10	8	0	1	1/14	10	1	1	2	19	10	3	4
Enterotoxigenic *E. coli,* < 50% haemolytic colonies (9*^b^)	5	4	1	1	0/4	4	0	0	0	6	4	0	2
Enterotoxigenic *E. coli,* > 50% haemolytic colonies (11*)	6	4	0	1	0/5	3	0	1	0	8	3	0	2
Enterotoxigenic *E. coli,* all (20*^c^)	11	8	1	2	0/9	7	0	1	0	14	7	0	4
Porcine Circovirus type 2 (5)	2	2	0	0	1/2	3	0	0	1	4	3	0	0
Rotavirus A (69^d^)	43	27	3	10	5/33	32	0	2	1	54	32	1	10
*Salmonella* spp (6)	4	3	0	1	1/3	3	0	0	0	5	3	0	1

For each pathogen, the total number of positive pigs is listed in the parenthesis in the first column. Moreover, the numbers of pigs with specific lesions within the different intestinal regions are presented for each of the pathogens

n: Number of positive pigs

*Seven animals were excluded due to unknown ETEC status

^a^Two of the 24 pigs had missing values in several of the variables due to autolysis

^b^Two of the nine pigs had missing values in several of the variables due to autolysis

^c^Two of the 20 pigs had missing values in one or more of the variables due to autolysis

^d^Four of the 69 pigs had missing values in one or more variables due to autolysis

### Gross pathology

Prevalence (%) of gross pathology among pigs with PWD and controls and the ORs with associated 95% CIs are shown in Table 3. Gastric ulcers had a lower OR in pigs with PWD as compared to pigs without PWD (OR 0.2, 95% CI 0.0; 0.7, Table 3). A total number of 105 pigs presented hyperkeratosis, gastric ulcers or both in the non-glandular compartment of the stomach. A higher prevalence of gastric ulcers, and stomachs with both ulcers and hyperkeratosis, respectively, were observed in the control group. Pigs with PWD had higher odds of abnormal content in the colon compared to pigs without PWD (OR 6.5, 95% CI 3.2; 14.3, Table 3).

**Table 3:** Prevalence of gross pathology among pigs with post-weaning diarrhoea (PWD) and controls, respectively, and the odds ratio (OR) of findings in pigs with PWD compared to pigs without PWD

Gross pathology	Prevalence PWD-cases (%)	Prevalence controls (%)	OR (95%CI)
Normal nutritional status	81	85	–
Lean nutritional status	18	15	1.2 (0.5;2.7)
*Stomach*			
No ulcer	43	32	–
Ulcer	3	13	0.2 (0.0;0.7)*
Hyperkeratosis	38	30	1 (0.5;2)
Ulcer and hyperkeratosis	13	24	0.4 (0.2;1.0)
*Jejunum*			
Normal content	48	57	NA **
Abnormal content	52	40	
No content	0	3	
No thickening	97	89	–
Thickened intestinal wall	2	4	0.6 (0.1;4.0)
Abnormal mucosa	0	0	–
*Ileum*			
Normal content	35	42	–
Abnormal content	10	5	2.5 (0.7;10.2)
No content	55	54	1.2 (0.7;2.3)
No thickening	90	93	–
Thickened intestinal wall	10	7	1.5 (0.5;4.6)
Normal mucosa	98	96	0.6 (0.1;4.3)
Abnormal mucosa	2	4	
*Colon*			
Normal content	13	51	–
Abnormal content	85	49	6.5 (3.2;14.3)*
Thickened intestinal wall	0	0	–
Normal mucosa	96	98	–
Abnormal mucosa	3	2	1.4 (0.2;12.4)

*95% CI not including one were considered statistically significant

**OR not calculated due to one group including 0

### Histopathology

**Fig. 1:**
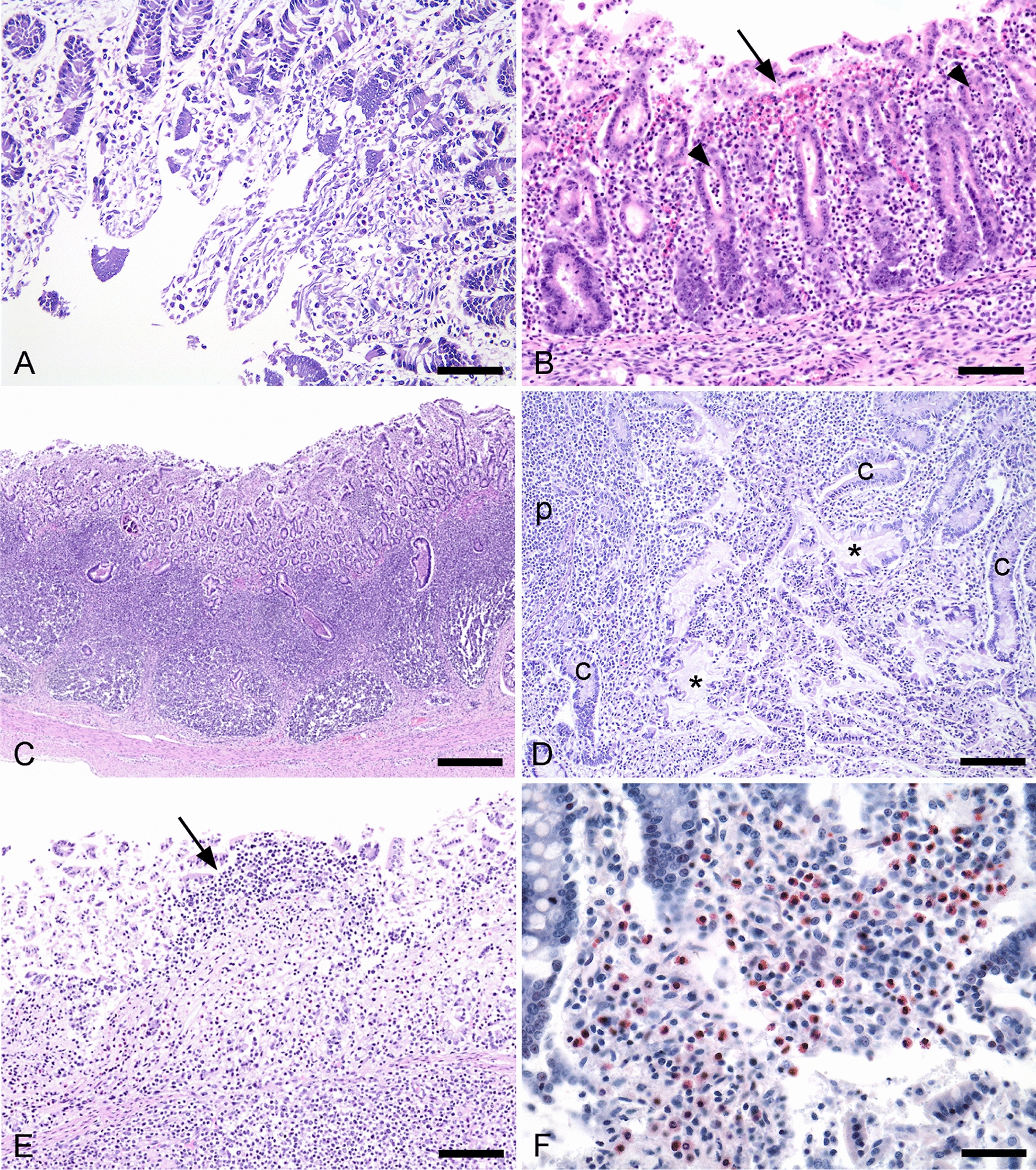
Histopathological findings in pigs with (**B**, **D**) and without (**A**, **C**, **E**, **F**) post-weaning diarrhoea. **A** Marked epithelial autolysis with loss of cellular details and architecture (20×/0.50 NA, scale bar, 250 µm. **B** Haemorrhage (arrow) in the lamina propria and crypt abscesses (arrowhead) in the colon (40×/0.95 NA, scale bar, 250 µm). **C** Hyperplastic crypts with abscess formation invading Peyer’s patches in the ileum (40×/0.95 NA, scale bar, 350 µm). **D** Dilated crypts (c) and hypersecretion (*) near Peyer’s patch (p) in the ileum (10×/0.30 NA, scale bar, 300 µm). **E** Focal mononuclear cell infiltration (arrow) in the lamina propria of ileum (40×/0.95 NA, scale bar, 250 µm). **F** Luna stain showing red cytoplasmic granules within eosinophilic granulocytes in the ileum (40x/0.75 NA, scale bar, 120 µm)

Histologically, various degrees of autolytic changes of the epithelium and parts of *lamina propria* precluded the ability to detect oedema, villus atrophy, and evaluate the epithelium for lesions such as goblet cell reduction and vacuolisation (Fig. 1A).

Hyperplastic crypts were observed in six of the jejuna (two with PWD), 93 of the ilea (45 with PWD), and 144 of the colons (78 with PWD). Eosinophilic infiltration was observed in 76 jejuna (35 with PWD), 134 of the ilea (69 with PWD) and in one colon which was from a control pig. A large inter-individual variation of the extent of infiltration was observed. Crypt abscesses were observed in 130 pigs (64 with PWD) in one or more of the intestinal regions (Figs. 1B, C). A total of 28 pigs presented neither crypt abscesses nor other lesions associated with inflammation such as neutrophilic granulocyte infiltration, haemorrhage or necrosis (in any intestinal region), of which 18 were cases with PWD. *Balantidium coli* was detected in the colon of 15 animals of which 8 were cases with PWD. Deposition of fibrin was only detected in one pig, which had diarrhoea, and was observed in relation to necrotic tissue in the lamina propria of the ileum.

**Fig. 2:**
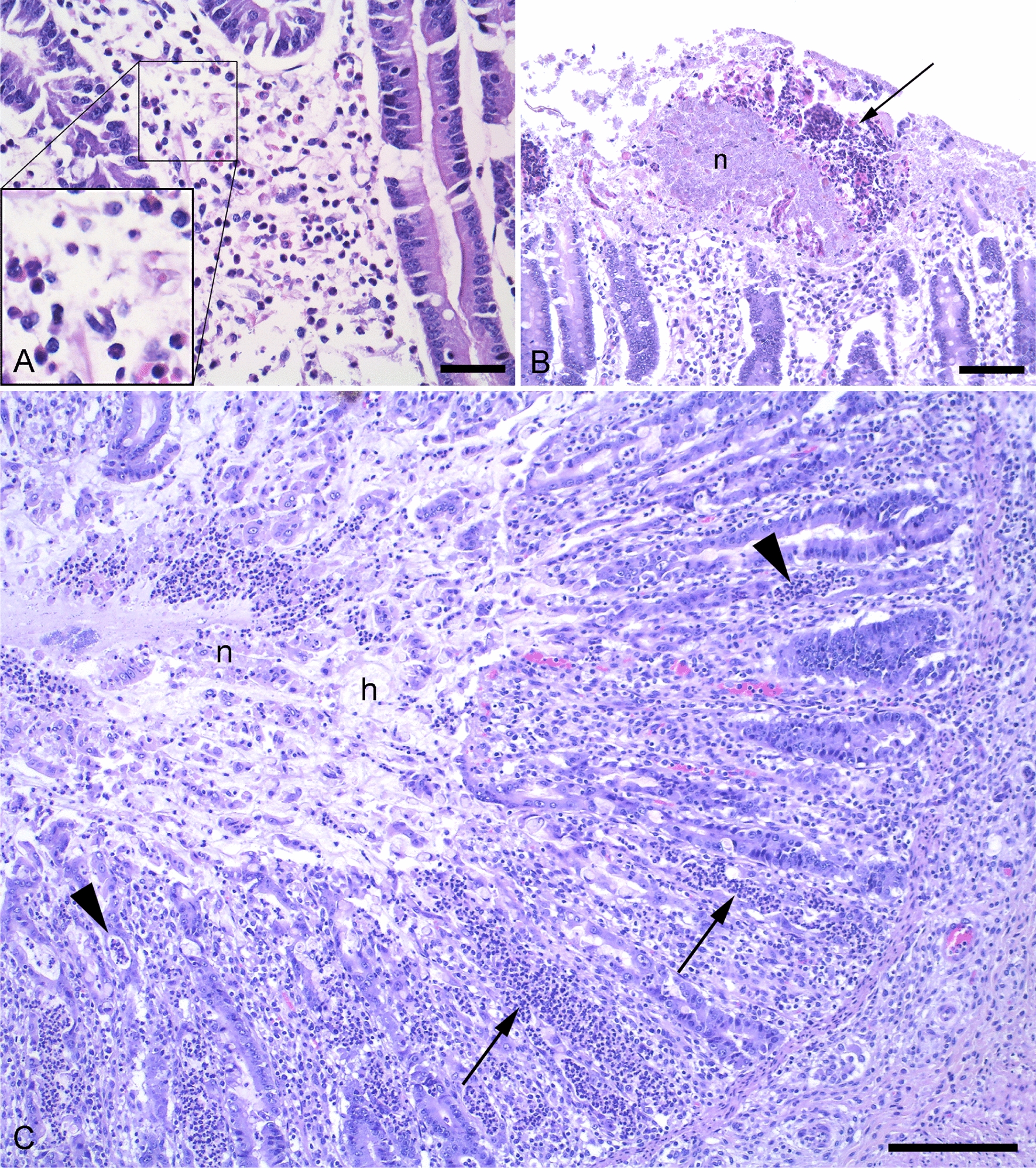
Histological signs of inflammation in pigs with (B) and without (A + C) post-weaning diarrhoea. Haematoxylin and eosin stains. **A** Neutrophilic granulocyte infiltration in the lamina propria of the jejunum (40×/0.75 NA, scale bar, 120 µm). Insert: Close-up of neutrophilic granulocytes. **B** Epithelial necrosis (n) partly bordered by neutrophilic granulocytes (arrow) in the colon (20×/0.50 NA, scale bar, 250 µm). **C** Colitis characterised by extensive neutrophilic granulocyte infiltration in the lamina propria (arrows), crypt abscesses (arrowheads), epithelial necrosis (n) infiltrated with neutrophilic granulocytes and hypersecretion (h) (10×/0.30 NA, scale bar, 200 µm)

**Fig. 3:**
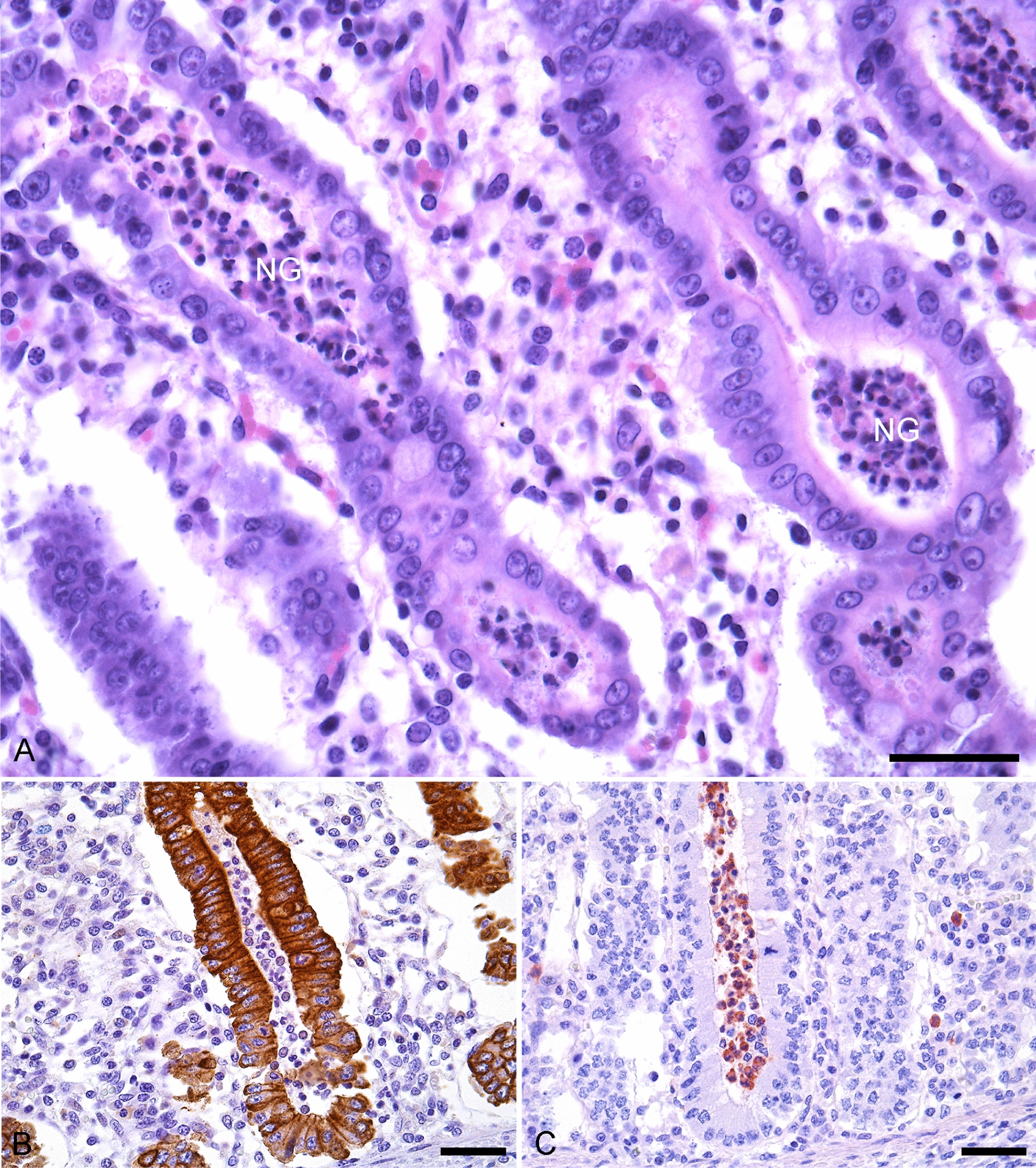
Crypt abscesses in pig with post-weaning diarrhoea. **A** Haematoxylin and eosin stain demonstrating neutrophilic granulocytes (NG), macrophages and debris in the lumen of the crypts (40×/0.75 NA, scale bar, 70 µm). **B** Cytokeratin stain demonstrating red/brown epithelial lining cells of the crypt abscess (40×/0.75 NA, scale bar, 120 µm). **C** MAC387 stain demonstrating red MAC-positive cells within the lumen of a crypt (40×/0.75 NA, scale bar, 120 µm)

The ORs with associated 95% CIs of crypt abscesses, dilated crypts, eosinophilic granulocyte infiltration, haemorrhage, hyperaemia, hyperleukocytosis, hyperplastic crypts, hypersecretion, mononuclear cell infiltration, necrosis, and neutrophilic granulocyte infiltration for pigs with PWD compared to controls are shown in Table 4. The representative lesions are shown in Figs. 1, 2 and 3A. Only neutrophilic granulocyte infiltration in the jejunum was significantly associated with PWD. Pigs with PWD had a lower odds of neutrophilic granulocyte infiltration (OR 0.3, 95% CI 0.1; 0.7, Table 4).

**Table 4:** The odds ratio (OR) of histopathological findings among pigs with post-weaning diarrhoea (PWD) compared to pigs without PWD

Histopathological findings	Jejunum OR (95%CI)	Ileum OR (95%CI)	Colon OR (95%CI)
Crypt abscesses (2/abundant)	NA**	4.4 (0.7;118.8)	1.9(0.7;5.3)
Dilated crypts	NA**	1.3 (0.7;2.5)	0.7 (0.4;1.3)
Eosinophilic granulocyte infiltration	0.7 (0.4;1.3)	1.0 (0.5;2.1)	NA**
Haemorrhage	NA**	NA**	0.5 (0.0;6.3)
Hyperaemia	1.2 (0.4;3.3)	3.8 (0.9;28.5)	1.6 (0.6;4.3)
Hyperleukocytosis	2.4 (0.8;8.1)	1.4 (0.7;3.2)	1.6 (0.4;8.4)
Hyperplastic crypts	0.5 (0.1;2.7)	0.8 (0.4;1.4)	1.4 (0.6;3.4)
Hypersecretion	NA**	1.3 (0.7;2.7)	0.7 (0.4;1.3)
Mononuclear cell infiltration	0.9 (0.3;2.5)	1.0 (0.5;2.3)	1.0 (0.4;2.4)
Necrosis	NA**	0.6 (0.1;4.3)	0.5 (0.0;6.3)
Neutrophilic granulocyte infiltration	0.3 (0.1;0.7)*	0.9 (0.4;2.3)	0.9 (0.4;2.2)

*95% CI not including one were considered statistically significant

**Insufficient information to estimate the odds ratio

The association between neutrophilic granulocyte infiltration in jejunum and PWD was different between the herds (P = 0.03). The difference was caused by an uneven distribution of pigs in four of the herds: In three herds, all pigs with neutrophilic granulocyte infiltration (n = 3, n = 1 and n = 8) were from the control group. Moreover, in one herd, neutrophilic granulocyte infiltration was seen in only a single case pig. Likewise, the associations between PWD and hyperleukocytosis (P = 0.04) and infiltration of eosinophilic granulocytes (P = 0.04) in ileum, respectively, were also herd dependent. Regarding hyperleukocytosis, all pigs with hyperleukocytosis in one herd (n = 4) were from the control group. Moreover, in three herds, all pigs with hyperleukocytosis (n = 2, n = 1 and n = 2) had PWD. For eosinophilic granulocyte infiltration, the difference was due to three herds where all of the pigs without eosinophilic granulocyte infiltration presented PWD (n = 1, n = 3 and n = 3). The distribution of pigs with diarrhoea and eosinophilic granulocyte infiltration appeared to be somewhat similar for the various herds.

The association between PWD and hyperplastic crypts and crypt abscesses, respectively, was not significantly different between herds in any of the intestinal regions (P > 0.05). Similarly, for neutrophilic granulocyte infiltration in ileum and colon, and for hyperleukocytosis and eosinophilic granulocyte infiltration in jejunum and colon, no interaction was present.

Histopathological manifestations in the jejunum, ileum and colon for the various pathogens are given in Tables 1, 2 and 5 for pigs with and without PWD, respectively. No specific pattern of lesions or apparent associations were observed for any of the pathogens or combination of pathogens. The same tendencies were observed, e.g., the majority of the pigs presented hyperplastic crypts in the colon and eosinophilic granulocyte infiltration in the ileum, for the various groups of pathogens.

**Table 5:** Histopathological manifestations in the jejunum, ileum and colon and the various combinations of pathogens in pigs with and without post-weaning diarrhoea (PWD)

Histopathology Pathogen (*n*)	Jejunum (*n*)									Ileum (*n*)				
	Crypt abscesses (abundant/few)	Dilated crypts	Eosinophilic granulocyte infiltration	Haemorrhage	Hyperleukocytosis	Hyperplastic crypts	Hypersecretion	Necrosis	Neutrophilic granulocyte infiltration	Crypt abscesses (abundant/few)	Dilated crypts	Eosinophilic granulocyte infiltration	Haemorrhage	Hyperleukocytosis
*Cases with PWD*														
Haemolytic *E. coli* (non ETEC) and rotavirus A (18*)	0/2	0	7	0	2	0	0	0	0	0/9	6	15	1	4
ETEC (> 50% haemolytic colonies) and rotavirus A (18^a^)	0/3	0	5	0	4	0	0	0	3	2/4	9	14	0	6
*Controls without PWD*														
Haemolytic *E. coli* (non ETEC) and rotavirus A (13)	0/1	0	6	0	0	2	0	0	0	0/7	4	9	0	0
ETEC (> 50% haemolytic colonies) and rotavirus A (8^b^)	0/0	0	2	0	1	0	0	0	3	0/8	2	6	0	2

Histopathology Pathogen (*n*)	Ileum (*n*)				Colon (*n*)								
	Hyperplastic crypts	Hypersecretion	Necrosis	Neutrophilic granulocyte infiltration	Crypt abscesses (abundant/few)	Dilated crypts	Eosinophilic granulocyte infiltration	Haemorrhage	Hyperleukocytosis	Hyperplastic crypts	Hypersecretion	Necrosis	Neutrophilic granulocyte infiltration
*Cases with PWD*													
Haemolytic *E. coli* (non ETEC) and rotavirus A (18*)	8	6	0	1	1/8	5	0	0	2	15	5	0	2
ETEC (> 50% haemolytic colonies) and rotavirus A (18^a^)	11	9	2	3	5/10	8	0	0	1	17	7	0	3
*Controls without PWD*													
Haemolytic *E. coli* (non ETEC) and rotavirus A (13)	6	4	0	0	0/6	4	0	1	0	10	4	1	2
ETEC (> 50% haemolytic colonies) and rotavirus A (8^b^)	4	2	0	0	0/4	2	0	1	0	6	2	0	2

For each combination of pathogens, the total number of positive pigs is listed in the parenthesis in the first column. Moreover, the numbers of pigs with specific lesions within the different intestinal regions are presented for each of the pathogens

ETEC: Enterotoxigenic* E. coli*

n: Number of positive pigs

*Two of the pigs had missing values in one or more of the variables due to autolysis

^a^One of the pigs had missing values in one of the variables (dilated crypts in colon) due to autolysis

^b^One of the pigs had missing values in several of the variables in jejunum due to autolysis

### IHC

Cytokeratin immunostained sections demonstrated red/ brown epithelium cells lining the crypts (Fig. 3B). The stains showed no necrotic epithelium in the crypt lumen. The MAC387 stained sections revealed MAC-positive cells, i.e., neutrophilic granulocytes and macrophages, in the crypt lumen (Fig. 3C).

All tissue sections stained with IHC towards PCV2 and *L. intracellularis* were negative.

## Discussion

The present study demonstrated a substantial number of gross and especially histopathological changes in weaned pigs with and without diarrhoea. Results from the necropsies showed animals to have abnormal (watery/slimy) content in the colon when diagnosed with PWD. This was expected, as these changes are commonly associated with diarrhoea [31]. However, as 13% of the animals with PWD had normal content in the colon, the absence of the change did not preclude diarrhoea (Table 3).

A higher prevalence of gastric lesions was observed in pigs without PWD and the odds of gastric ulcers was lower among pigs with PWD compared to pigs without PWD (Table 3). Gastric lesions are prevalent in finisher pig herds and the aetiology considered multifactorial [8, 32]. Infectious aetiologies have been suggested [8, 33], and risk factors include both particle-size in feed and management [32, 33]. Interestingly, more than half of the pigs in this study presented hyperkeratosis, gastric ulcers or both (n = 105), which may constitute a welfare and production problem [34]. This is in accordance with a previous study of ulcers in the non-glandular part of the stomach in nursery pigs [32].

Advanced autolytic changes in the gastro-intestinal tissues can be expected when transit time exceeds an hour (or less) [35]. However, the approximately 24 h transit time from euthanasia to necropsy in this study is a realistic timeframe for pigs submitted to diagnostic necropsy in Denmark. For optimal histological visualisation of the tissues, formalin fixation of the intestines should be done on site/in the herds.

Crypt abscesses were found in most of the pigs (n = 130) and both in pigs with and without clinical PWD. Though the OR of the lesion was non-significant, an abundant number of crypt abscesses in ileum and colon, respectively, was more frequently observed in the groups with PWD (Tables 1, 2 and 5), regardless of aetiology. *S. typhimurium* (in a co-infection with *Entamoeba polecki*) [36] and *Serpulina pilosicoli* (*B. pilosicoli*) [37] have previously been associated with the presence of crypt abscesses and enteritis/colitis in pigs. In addition, PCV2 has also been linked to crypt lesions in the colon [38], somewhat similar in appearance to those of this study. However, in that study the content of the crypts was considered to be necrotic debris (epithelial cell necrosis) in contrast to the lesions in the present study, where cytokeratin and MAC387 stains confirmed the lesions to be crypt abscesses (Fig. 3). Though all pigs infected with *Salmonella* spp. and PCV2 presented crypt abscesses in one or more intestinal region(s), crypt abscesses were also found in animals infected with rotavirus A, haemolytic *E. coli* (non-ETEC), ETEC, *C. perfringens* as well as in animals with negative microbiological outcome, suggesting that the lesion is not related to a specific aetiology in this study.

Hyperplastic crypts were commonly present in the colon and ileum of pigs with and without PWD, and there was no significant association to diarrhoea (Table 4). The prevalence of the manifestation was slightly similar/balanced for the various pathogens shown in Tables 1 and 2. Hyperplastic crypts have previously been considered a normal finding, i.e., porcine lymphoglandular complexes consisting of epithelial crypts invading the lymphoid nodules [39]. Others have shown *L. intracellularis* to cause epithelial hyperplasia in pigs [15, 40, 41]. However, *L. intracellularis* was not demonstrated by qPCR or IHC in any of the pigs. Hyperplastic crypts in the colon have also been speculated to be a result from protein fermentation [7, 42], and in a previous study it was demonstrated that an increase in crude protein in five-week-old pigs was associated with diarrhoea [43].

The inter-individual variation in the quantity of eosinophilic granulocytes was high, and results show no associations of eosinophilic granulocyte infiltration to PWD or specific pathogens. The association between diarrhoea and eosinophilic granulocyte infiltration in ileum appeared to be herd-dependent. However, this might be a random finding (statistical Type I error). As it appeared there were only little differences between the herds in regard to the prevalence of diarrhoea among pigs with eosinophilic granulocyte infiltration in ileum, environmental impact and differences in feed (type 1 hypersensitivity reaction) seemed to be dubious causes. Parasites are also known to cause infiltration of eosinophilic granulocytes [8, 44]. However, *B. coli,* which is commonly found as a commensal in the large intestines [8], was the only parasite detected and only in 15 animals. As eosinophilic granulocyte infiltrations occurred in one colon, 134 ilea and 76 jejuna, it appears highly unlikely that the parasite is the cause of the lesion.

The odds of neutrophilic granulocyte infiltration were significantly lower in the jejunum among pigs with PWD compared to pigs without PWD (Table 4). As neutrophilic granulocytes are phagocytes and the first type of leukocyte in inflammatory responses [45], the result appeared counter-intuitive. In addition, neutrophilic granulocytes association to PWD was different between the herds for jejunum. However, as only 27 pigs had neutrophilic granulocyte infiltration in jejunum, of which seven had diarrhoea, the results are considered a random finding. The herd-dependent association between PWD and hyperleukocytosis was borderline significant (P = 0.04). As this was based on findings in few pigs, the difference in the association might also have attributed to random error. However, the various herds might have different causative aetiologies or other underlying factors that might cause this difference in association.

A rather high prevalence of lesions in general was observed, e.g., crypt abscesses (n = 130), hyperplastic crypts in the colon (n = 144) and eosinophilic granulocyte infiltration in the ileum (n = 134). This reveals a general negative gut health condition, which may be related to the early age at weaning.

There are different challenges in the design of these types of studies. Therefore, this study was primarily of descriptive nature. Often, a broad study population (as possible) is wanted to make this representative of the target population, and to include the most likely pathogens and managerial factors encountered. However, the study should also be feasible in terms of sample size and logistics; therefore, an appropriate number of herds were included. The consequences are clustering of effects within herds, and restricted sample sizes per herd. Such design issues should in principle be taken into account, but a random effect of herd in a statistical model presumes that this is normally distributed. A fixed effect in a statistical model would be the alternative, but the sample size cannot support this. Therefore, the study was kept as a primarily descriptive one and it was not possible to determine if the herd effect was a confounding factor.

As the clinical evaluations were done on a single day in each herd, it is also important to remember that the results are ‘snapshots’ of the condition in the herds. Pigs with lesions may be in ‘spontaneous recovery’ without treatment, i.e., might not have eaten for a while, or perhaps would develop diarrhoea and/or lesions the very next day. In addition, screening has only included known specific pathogens and the swabs used for microbiology were transported in the same medium (PBS) to the laboratory, which may affect their survival differently. Therefore, longitudinal supplementary studies including analyses of dietary impact on the intestinal microbiome as well as investigations of new potential causative/infectious pathogens could be of great value.

## Conclusions

The variation between herds might be due to unidentified pathogens, differences in environmental impact or feed. No apparent association was found between lesions and the various pathogens or a combination/interaction of these. In addition, histopathology revealed several lesions not associated with PWD, and some pigs with diarrhoea did not present signs of inflammation. The present study emphasises that the association between especially histopathological manifestations and specific pathogens and PWD, respectively, is more complex than anticipated.

## Supplementary Information

**Additional file 1: Table S1**. Pathogens, assay names, and primer and probe sequences used for detection of bacteria and virus in the high-throughput real-time PCR analysis.

## Data Availability

The datasets used and/or analysed during the current study are available from the corresponding author on reasonable request.

## Abbreviations

CI: Confidence intervals
ETEC: Enterotoxigenic *Escherichia coli*
HE: Haematoxylin and eosin
IHC: Immunohistochemistry
MALDI-TOF: Matrix-assisted laser desorption ionisation time-of-flight
OR: Odds ratio
PWD: Post-weaning diarrhoea
qPCR: Quantitative polymerase chain reaction
Real-time PCR: Real time polymerase chain reaction
RT-PCR: Reverse transcriptase polymerase chain reaction
TBS: Tris-buffered saline
