# Supplementary material for: Gastro-intestinal lesions are not relatable to diarrhoea or specific pathogens in post-weaning diarrhoea (PWD) in pigs
Source: Acta Vet Scand. 2023 Jul 3;65:30. doi: 10.1186/s13028-023-00693-y (PMC10316552; doi:10.1186/s13028-023-00693-y)
Supplement: Supplementary file 1 — Additional file 1: Table S1. Pathogens, assay names, and primer and probe sequences used for detection of bacteria and virus in the high-throughput real-time PCR analysis. [file 13028_2023_693_MOESM1_ESM.docx]

**Supplementary Table 1. Pathogens, assay names, and primer and probe sequences used for detection of bacteria and virus in the high-throughput real-time PCR analysis.**

| **Pathogen** | **Target gene** | **Name** | **Sequence (5’-3’)** | **Length (bp)** | **Reference** |
| --- | --- | --- | --- | --- | --- |
| *B. pilosicoli* | 23S rRNA | B.pilo-F  B.pilo-R  B.pilo-P | GTAGTCGATGGGAAACAGGT  TTACTCACCACAAGTCTCGG  FAM-TATTCGACGAGGATAACCATCACCT-BHQ1 | 124 | [24] |
| *L. intracellularis* | 16S rRNA | Law-F  Law-R  Law-P | GCGCGCGTAGGTGGTTATAT  GCCACCCTCTCCGATACTCA  FAM-CACCGCTTAACGGTGGAACAGCCTT-TAMRA | 98 | [24] |
| *E. coli* type F4 | *faeG* | E.coli F4-F  E.coli F4-R  E.coli F4-P | CACTGGCAATTGCTGCATCT  ACCACCGATATCGACCGAAC  FAM-TCACCAGTCATCCAGGCATGTGCC-TAMRA | 86 | [24] |
| *E. coli* type F18 | *fedA* | E.coli F18-F  E.coli F18-R  E.coli F18-P | GGCGGTTGTGCTTCCTTGT  CCGTTCACGGTTTTCAGAGC  FAM-TAACTGCCCGCTCCAAGTTATATC AGCTGTT-TAMRA | 128 | [24] |
| Rotavirus A | NSP3 | Rota A-F  Rota A-R  Rota A-P | ACCATCTACACATGACCCTC  GGTCACATAACGCCCC  FAM-ATGAGCACAATAGTTAAAAGCTAACACTGTCAA-TAMRA | 87 | [24] |
| Porcine circovirus type 2 | CAP | PCV2-F  PCV2-R  PCV2-P | GATGATCTACTGAGACTGTGTGA  AGAGCTTCTACAGCTGGGACA  FAM-TCAGACCCCGTTGGAATGGTACTCCTC-BHQ1 | 152 | [24] |
